# Effects of Genetic Polymorphisms of Cathepsin A on Metabolism of Tenofovir Alafenamide

**DOI:** 10.3390/genes12122026

**Published:** 2021-12-20

**Authors:** Soichiro Ito, Takeshi Hirota, Miyu Yanai, Mai Muto, Eri Watanabe, Yuki Taya, Ichiro Ieiri

**Affiliations:** 1 Department of Clinical Pharmacokinetics, Graduate School of Pharmaceutical Sciences, Kyushu University, Fukuoka 812-8582, Japan; soichiro.ito@jt.com (S.I.); miyuu_0221@yahoo.co.jp (M.Y.); mai.lemon96@gmail.com (M.M.); watanabe.eri.pk@gmail.com (E.W.); 2 Drug Metabolism and Pharmacokinetics Research Laboratories, Central Pharmaceutical Research Institute, Japan Tobacco Inc., Osaka 569-1125, Japan; yuki.taya@jt.com; 3 Pharmacy, Kyushu University Hospital, Fukuoka 812-8582, Japan; thirota@phar.kyushu-u.ac.jp

**Keywords:** cathepsin A, genetic polymorphisms, tenofovir alafenamide

## Abstract

Cathepsin A (CatA) is important as a drug-metabolizing enzyme responsible for the activation of prodrugs, such as the anti-human immunodeficiency virus drug Tenofovir Alafenamide (TAF). The present study was undertaken to clarify the presence of polymorphisms of the CatA gene in healthy Japanese subjects and the influence of gene polymorphism on the expression level of CatA protein and the drug-metabolizing activity. Single-strand conformation polymorphism method was used to analyze genetic polymorphisms in healthy Japanese subjects. Nine genetic polymorphisms were identified in the CatA gene. The polymorphism (85_87CTG>-) in exon 2 was a mutation causing a deletion of leucine, resulting in the change of the leucine 9-repeat (Leu9) to 8-repeat (Leu8) in the signal peptide region of CatA protein. The effect of Leu8 on the expression level of CatA protein was evaluated in Flp-In-293 cells with a stably expressed CatA, resulting in the expression of CatA protein being significantly elevated in variant 2 with Leu8 compared with Leu9. Higher concentrations of tenofovir alanine (TFV-Ala), a metabolite of TAF, were observed in the Leu8-expressing cells than in the Leu9-expressing cells using LC/MS/MS. Our findings suggest that the drug metabolic activity of CatA is altered by the genetic polymorphism.

## 1. Introduction

Prodrugs have played a very important role in drug development. Thirty-one of the 249 medicines approved by the Food and Drug Administration during the decade from 2017 to 2008 were pro-drugs. Nearly two prodrugs were approved each year during the decade except for 2016 [1]. The prodrugs were metabolized into active forms with pharmacological activity. Many metabolizing enzymes, such as carboxylesterase (CES), are involved in metabolic activation, and the effect of the polymorphism of these metabolizing enzymes on the drug metabolic activity is very important from the viewpoint of the appropriate use of the drug [2]. Cases in which the genetic polymorphism of the drug-metabolizing enzyme influenced the pharmacokinetics of the prodrug are shown below. Dabigatran etexilate, a prodrug with improved bioavailability of dabigatran, is activated by the hepatic drug-metabolizing enzyme CES1, and exhibits an antithrombin effect. Previous studies showed that patients with a single nucleotide polymorphism (SNP) of CES1 had lower blood levels of dabigatran and a lower risk of bleeding as a side effect compared with the wild type [3]. The pharmacokinetics and pharmacological effects of tramadol, a known prodrug, were affected by cytochrome P450 2D6 (CYP2D6) gene polymorphism. The effects of the variants in CYP2D6 were found to be attenuated in poor metabolizers (PM) and enhanced in ultrafast metabolizers (UM) [4]. The incidence of PM in the Japanese population is extremely low, at less than 1.0%, compared with that in Western populations (5~10%), suggesting that CYP2D6 genetic polymorphisms have a limited impact on the Japanese population [5]. Clopidogrel is a prodrug activated by CYP2C19, and CYP2C19 polymorphisms were reported to affect the metabolism of clopidogrel [6]. Eighteen to 22.5% of the Japanese population show decreased CYP2C19 activity, suggesting that the antiplatelet effect of clopidogrel in the Japanese population varies among individuals [6,7]. There are large individual differences in the metabolic enzymes involved in the conversion of the prodrug to its active form. It is important for personalized drug therapy to identify genetic factors that contribute to individual differences in drug-metabolizing activity.

**Figure 1:**
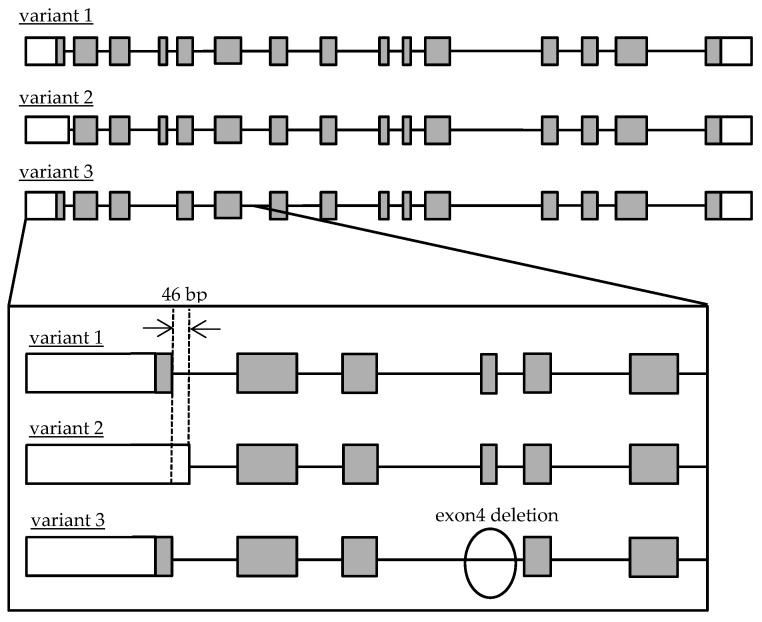
Schematic diagram of genomic Cathepsin A variant 1, 2 and 3 structures. Open and gray boxes represent untranslated and translated regions.

Recently, it was clarified that Cathepsin A (CatA) plays an important role in the metabolic activation of prodrugs such as Tenofovir Alafenamide (TAF/GS-7340), which has been used in antiretroviral therapy for human immunodeficiency virus (HIV) and hepatitis B and Sofosbuvir (SOF/GS-7977/PSI-7977) for hepatitis C virus [8,9]. The importance of CatA as a drug-metabolizing enzyme has been recognized in the research and development of drugs and appropriate use of medicine [8]. CatA is a multifunctional glycoprotein mainly distributed in lysosomes. CatA undergoes several steps to produce a mature protein. First, it is translated as a preprotein and transported to the endoplasmic reticulum, where the signal peptide is cleaved and an N-type sugar chain is added to form a 54-kDa precursor. Subsequently, an approximately 3-kDa polypeptide is removed and converted to mature 31 and 20-kDa forms [10]. CatA is widely expressed in the liver, kidney, and lung [11], and was also found in peripheral blood mononuclear cells (PBMC) including CD4 + T cells that are HIV target cells [12]. CatA has a protective function such as the stabilization of β-galactosidase and activation of neuraminidase [13,14], and an enzyme function, such as acid carboxypeptidase, neutral esterase, and deamidase [15]. It was reported that an abnormality of CatA caused the galactosialidosis (GS) of autosomal recessive genetic disease, which is a type of lysosomal disease. To date, 36 mutations of the CatA gene have been reported [16]. Genetic analysis of the CatA gene in GS patients has been performed, but the frequency and function of the genetic polymorphisms in healthy adults have not been clarified. Three transcript variants of CatA were reported in the National Center for Biotechnology Information (NCBI) Reference Sequence Database (Refseq), but no major variants have been identified in humans [17,18]. The accession numbers are variant 1 (NM_000308.3 → NP_000299.2), variant 2 (NM_001127695.2 → NP_001121167.1), and variant 3 (NM_00116759.4.2 → NP_001161066.1). The gene structures are shown in Figure 1.

Caciotti et al. decided to use variant 1 as a reference sequence (wild) based on the guidelines of the Human Gene Mutation Database (HGMD) to prevent confusion regarding reporting of gene mutations [19]. In this study, we focused on the major transcript variant of CatA in healthy Japanese subjects and investigated the genetic polymorphisms of CatA and their effects on mRNA, protein expression, and metabolic activity.

## 2. Materials and Methods

### 2.1. Quantitative Reverse Transcription-PCR Analysis

RNA was isolated and quantitative reverse transcription-PCR analysis of total RNA from buffy coats of five Japanese was conducted using the RNeasy plus Mini Kit (Qiagen, Hilden, Germany) according to the manufacturer’s instructions. The RNA samples were reverse-transcribed into first-strand cDNA with 2 μg of total RNA, 4.0 μL of 5× first strand buffer, 4.0 μL of 0.1 mM DTT, 1.0 μL of 500 μg/mL random primers (Promega, Madison, WI, USA), 4.0 μL of 10 mM dNTP mixture, and 100 units of SuperScript II RNase H-reverse transcriptase (Invitrogen Life Technologies, Carlsbad, CA, USA). The reaction mixture was incubated at s42 °C for 60 min. The mRNA level was measured with a real-time PCR system (Applied Biosystems, Foster City, CA, USA). The primers for CatA mRNA total, variant 1, and variant 2 are shown in Table 1.

The PCR amplicons of CatA total, variant 1, and variant 2 were purified and cloned into pGEM^®^-T Easy Vector (Promega). The reconstructed plasmids were transferred into E.coli JM 109 Competent Cells (NIPPON GENE, Tokyo, Japan) to obtain plasmids containing CatA- related genes. The plasmid was extracted using a commercially available kit (QuickLyse^®^ plasmid Kit, Qiagen). Quantitative Real-time PCR assay was performed on a StepOnePlus Real-time PCR System (Applied Biosystems) with ROX reference dye and SYBR^®^ Primer EX TaqTM (TaKaRa, Tokyo, Japan). A 10-fold dilution series of the standard plasmid for the related target was run with the samples to prepare a standard curve. The copy number of each sample was calculated based on the copy number of series of the standard plasmid.

### 2.2. PCR-SSCP Analysis

Blood samples were obtained from unrelated Japanese volunteers (76 subjects in each: Tennessee Blood Services, Memphis, TN, USA). DNA extraction from peripheral blood was performed with an automatic nucleic acid extraction device Toyobo HMX-2000 (Toyobo, Osaka, Japan). Primers were designed to analyze cording regions and 5′ FLR 600 bp in the CatA gene (GenBank accession number: NG-008291.1). Primer pairs were used for 35–40 cycles to amplify genomic DNA. The following conditions were used in each cycle: 95 °C for 40 s, 50–65 °C for 45 s, and 72 °C for 1 min. PCR products were analyzed with the single strand conformation polymorphism (SSCP) method to assess genetic variations, followed by sequencing. Sequencing was performed for PCR products directly or after subcloning on an ABI 3100 automatic sequencer (Applied Biosystems) using a Big-Dye Terminator Cycle Sequencing Ready Reaction Kit (Applied Biosystems).

### 2.3. Luciferase Reporter Assay

The 5′ FLR region of the CatA gene was amplified from human genomic DNA using forward and reverse primers (Forward: 5′-CAGATATGGTACCACGAGGAG-3′, Reverse: 5′-CTGGAAGTGATGTGTACGAGTC-3′). Kpn I (TaKaRa) restriction sites were created in the primers for cloning. The products were cloned into the pGL3 Promoter vector (Promega). K562 cells were plated in a 24-well plate at a density of 5.0 × 10^4^ cells/well and maintained in Opti-MEM with Lipofectamine™2000 (Invitrogen), pRL-TK, and the pGL3 Promoter vectors with 5′ FLR constructs of CatA gene at 37 °C in 5% carbon dioxide. After 24 h, reporter assays were performed using the Dual-Luciferase Reporter Assay System (Promega) and normalized for transfection efficiency by co-transfected Renilla-luciferase.

### 2.4. CatA-Stable Expressing Cells

Flp-In-293 cells (Invitrogen) were maintained in DMEM supplemented with 10% (*v/v*) heat-inactivated FBS (Sigma-Aldrich, St. Louis, MO, USA) in 5% (*v*/*v*) CO_2_. The Flp-In-293 cells were transfected with the pcDNA5/FRT/CatA expression vectors and the Flp recombinase expression plasmid pOG44 using LipofectAmine™ (Invitrogen). Colonies resistant to 100 µg/mL hygromycin B (Nacalai Tesque, Kyoto, Japan) were picked and sub-cultured. The picked cells were maintained in DMEM supplemented with 10% (*v*/*v*) heat-inactivated FBS, and 100 µg/mL hygromycin B in 5% (*v*/*v*) CO_2_.

### 2.5. Western Blotting

The lysate samples were separated on 13% SDS-polyacrylamide gels and transferred to polyvinylidene difluoride membranes (Millipore, MA, USA). The membranes were hybridized with a rabbit polyclonal antibody against human CatA (Rockland Immunochemicals, PA, USA) and mouse monoclonal antibody againstβ-actin (Abcam, Cambridge, UK). The immune complexes were hybridized with anti-rabbit IgG horseradish peroxidase-linked whole antibody donkey (GE Healthcare, Buckinghamshire, UK) and anti-mouse IgG horseradish peroxidase-linked whole antibody sheep (GE Healthcare), respectively. Membranes were washed three times in PBS-Tween, and specific bands were visualized using AmershamTM ECL PrimeTM Western blotting Analysis System and LAS-3000 (FUJIFILM, Tokyo, Japan) according to the manufacturer’s instructions.

### 2.6. LC/MS/MS Condition on the Detection of TFV-Ala

In order to confirm the drug metabolic activity of CatA, a system to measure the amount of TFV-Ala, the primary metabolite of HIV therapeutic agent TAF, a representative substrate, was constructed. The triple quadrupole UHPLC/MS/MS analyses of TFV-Ala as a metabolite of TAF and Daidzein as an internal standard were conducted on a Nexera/LCMS-8050 (Shimadzu, Kyoto, Japan) using solvent A of 0.2% formic acid in H_2_O and solvent B of MeCN/MeOH/IPA/H2O = 1/1/1/1 (*v*/*v*) and a reversed-phase column Mastro (C18, 3 μm, 2.1 × 150 mm, Shimadzu GLC, Tokyo, Japan). A linear gradient was used to separate the analytes: solvent B; 2% (0–1.5 min), 2% to 95% (1.5 to 5 min), 95% (5 to 6.5 min), 95% to 2% (6.5 to 6.6 min), 0% (6.6 to 8 min) at a flow rate of 0.4 mL/min and a column temperature of 35.0 °C. TFV-Ala and Daidzein were detected in ESI+ and multiple reaction monitoring mode (MRM) using the following mass transitions (TFV-Ala: *m*/*z* 359.0 > 270.0, Daidzein: *m*/*z* 255.1 > 199.1. Since the reagent of TFV-Ala was unavailable, a Q3 product scan was performed using the supernatant samples of CatA stably expressing cells incubated with 5 µM TAF for 60 min, and the analytical conditions were determined (Table 2).

### 2.7. CatA Metabolic Activity

The stably expressing CatA cells were incubated in DMEM with 5 µM TAF (Toronto Research Chemicals, North York, Canada) and 10% FBS at 37 °C for 60 min. After incubation, an equal volume of acetonitrile was added to the supernatant and finally diluted to the composition of 33% acetonitrile with 0.4 µM Daidzein (Sigma-Aldrich, St. Louis, MO, USA) as an internal standard for LC/MS/MS analysis.

## 3. Results

### 3.1. Quantification of Transcriptional Variants of CatA in Lymphocytes

**Figure 2:**
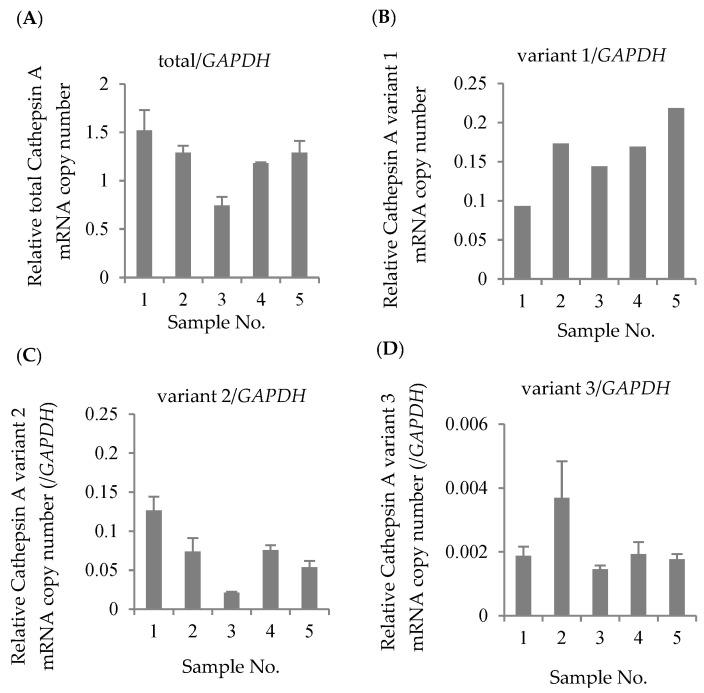
Absolute expression levels in Cathepsin A mRNA in Japanese lymphocytes. The copy numbers of total Cathepsin A variants (**A**), variant 1 (**B**), variant 2 (**C**), and variant 3 (**D**) were measured by real-time RT-PCR and were normalized by the copy number of *GAPDH*. The data represented as the mean ± S.D.

The mRNA expression levels of total CatA, variant 1, variant 2, and variant 3 were quantified by real-time PCR using 5 blood samples of healthy Japanese subjects. The housekeeping gene glyceraldehyde 3-phosphate dehydrogenase (GAPDH) was used as an internal standard. As a result of absolute quantification, expressions of variants 1–3 were observed in all samples (Figure 2). The results showed that variants 1 and 2 were expressed at approximately the same level, and that the expression level of variant 3 was significantly lower than those variants. Thus, the major transcripts of CatA in human lymphocytes were suggested to be variants 1 and 2.

### 3.2. Genetic Polymorphism of CatA Gene

Genomic DNA extracted from peripheral blood of 76 healthy Japanese subjects was used to investigate the genetic polymorphism of CatA. All 15 exons and 600 bp upstream of the translational start site (5′ FLR) of the human CatA gene, which has been reported to affect transcriptional activity [17], were analyzed. The genetic variants were screened by single-strand conformation polymorphism (SSCP) analysis, and the sequence of the mutation was identified by the Dye Terminator method. As a result, nine genetic polymorphisms were identified (Table 3). Three of these polymorphisms were located in 5′ FLR, three were in the exon, and the other three were located in the intron. The mutation (85_87CTG>-) in exon 2 caused the deletion of leucine (Leu), resulting in the change of the leucine 9-repeat (Leu9) to 8-repeat (Leu8) in the signal peptide region. Homozygotes (Leu8/Leu8) for 85_87CTG>- were most frequent in 29 of 48 Japanese subjects, followed by heterozygotes (Leu9/Leu8) and the wild type (Leu9/Leu9). The haplotypes were estimated by Arlequin V.3.5 based on the identified genotypes, and five haplotypes were identified (Table 4).

### 3.3. Effects of Polymorphisms of CatA Gene on Transcription Activity

**Figure 3:**
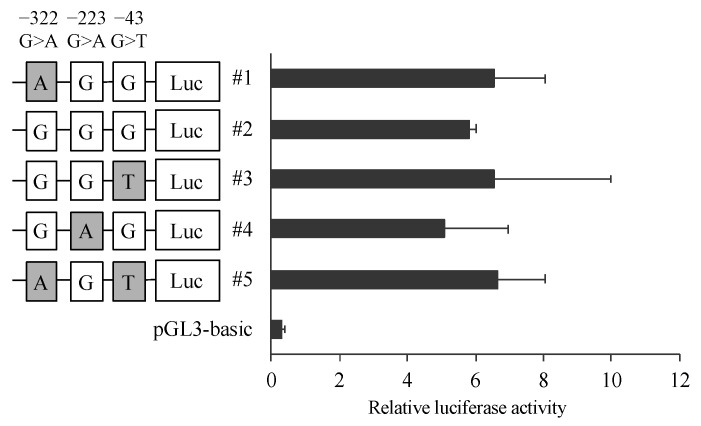
Reporter gene activities of Cathepsin A 5′-FLR. Luciferase reporter gene vectors containing each polymorphisms were transfected into the K562 cells. Relative luciferase activity of each reporter vector is Firefly luciferase activity normalized to Renilla luciferase activity. The data represented as mean + S.D. of triplicate experiments.

To clarify the effect of the variants in 5′ FLR on the transcriptional activity of the CatA gene, dual luciferase assays were performed. A reporter vector was constructed in which the 5′ FLR sequence of CatA was incorporated upstream of the luciferase gene of the pGL3-basic vector. Blood cell-derived K562 cells transfected with the reporter vector containing 5′ FLR showed higher transcriptional activity than the cells transfected with the control vector, but there was no significant difference in transcriptional activity between the five haplotypes (Figure 3).

### 3.4. Effects of Polymorphism of CatA Gene on Protein Expression

**Figure 4:**
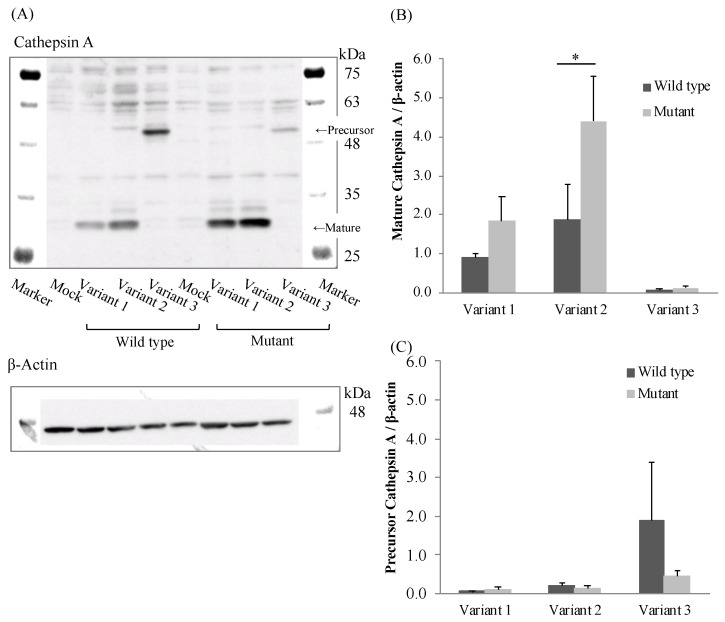
Western blotting analysis in stably Cathepsin A expressing Flp-in 293 cells. (**A**) Cathepsin A precursor, mature and β-actin. (**B**) Densitometric analysis was shown for precursor Cathepsin A/β-actin of the blots. (**C**) Densitometric analysis was shown for mature Cathepsin A/β-actin of the blots. The data represented as mean + S.D. of triplicate experiments. (*t* test * *p* < 0.05).

To evaluate the effect of the mutation (85_87CTG>-) in three transcriptional variants on CatA protein expression, we established CatA-stable expressing FLP-in 293 cells using the pcDNA5/FRT vector. CatA protein was quantified by Western blotting using protein extracted from the established cells (Figure 4A). As a result of comparing CatA protein expression levels among the variants, the expression level of mature CatA (31 kDa) tended to be higher in variant 2 than in variant 1 in all genotypes (Figure 4B). While variant 3 showed the strongest precursor CatA (54 kDa) expression among the variants, there was little expression of mature CatA. (Figure 4B,C). The expression of mature CatA in variant 2 was significantly higher in the mutant (Leu8) than in the wild type (Leu9) (*p* < 0.05) (Figure 4B). There was no significant effect of the mutant (Leu8) on the mature protein expression in variants 1 and 3. On the other hand, the expression level of the CatA precursor was not significantly affected by the mutant (Leu8) in any of the variants (Figure 4C). These results suggest that the mutation (85_87CTG>-) affects only the mature CatA in variant 2.

### 3.5. Effects of Polymorphism of CatA on Metabolic Activity

**Figure 5:**
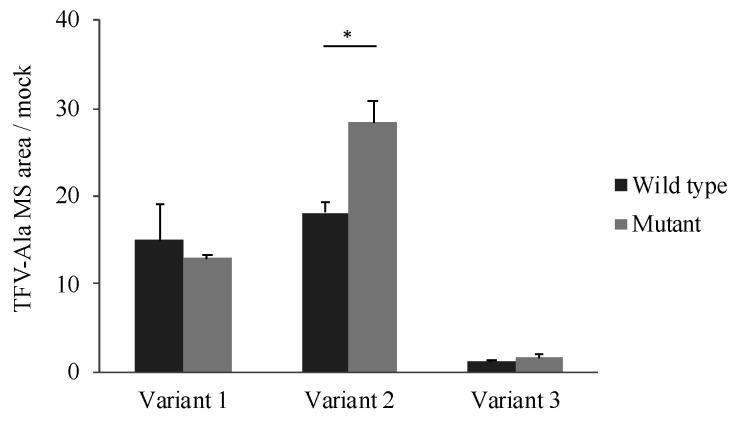
Metabolism of TAF to TFV-Ala in stably Cathepsin A expressing Flp-in 293 cells. Cells were incubated with 5 µM TAF for 60 min. TFV-Ala in supernatants was quantified by LC-MS/MS. The data represented as mean + S.D. of triplicate experiments. (*t* test * *p* < 0.05).

The effect of the polymorphism (85_87CTG>-) on CatA enzyme activity was examined. TAF, which has been reported as a specific substrate for CatA, was incubated for 60 min at a concentration of 5 μM in the cells stably expressing the CatA transcriptional variant with the polymorphism (85_87CTG>-). Tenofovir alanine (TFV-Ala), a metabolite of TAF, was measured by liquid chromatography/mass spectrometer (LC/MS/MS). In all variants, TFV-Ala was detected in CatA-stable expressing cells (Figure 5). The amount of TFV-Ala was markedly lower in the variant 3-transfected cells than that in variants 1 and 2. On the other hand, a significant increase in TFV-Ala was observed in variant 2 with the mutant (Leu8) compared with the wild type (Leu9) (Figure 5).

## 4. Discussion

In this study, we identified genetic polymorphism of the CatA gene in healthy Japanese subjects and analyzed the effects of the polymorphisms on the transcriptional activity, level of protein expression, and activity of drug metabolism. Although three transcript variants (1–3) have been reported for the CatA gene (Refseq: variant 1;NM_000308.3→NP_000299.2, variant 2; NM_001127695.2→NP_001121167.1, variant 3; NM_001167594.2→NP_001161066.1), the major transcript variant in human lymphocytes, the target site of TAF, remains unknown. We investigated the transcripts of CatA in healthy Japanese subjects and showed that the major transcripts of CatA in human lymphocytes were variants 1 and 2. Expression of variant 3 was significantly lower than that of the two other variants (Figure 2). Variant 3 lacked exon 4 and was 51 bases shorter (17 amino acids) than variants 1 and 2, suggesting a difference in metabolic properties.

Polymorphism analysis was performed in 5′ FLR (600 bp) and all exons of the CatA gene. Six polymorphisms were identified in the exons and three in the introns. There were 36 reports of CatA polymorphism in GS patients [19]. The polymorphisms identified in GS patients were not observed in the results of this study, because the subjects analyzed in this study were healthy. The polymorphisms in this study were given NCBI reference numbers (Table 3). dbSNP showed sequence information for the CatA genetic polymorphism but did not provide data on the frequency of genotypes or origin of the genome used for the analysis. In this study, we identified the genotype distribution in healthy Japanese subjects.

The 5′ FLR region analyzed in this study has been reported to exhibit promoter activity [17]. In addition, the binding sequence of the transcription factor EB, known as coordinated lysosomal expression and regulation, overlaps with the upstream transcription factor/major late transcription factor motif, and has been reported to be involved in expression of the CatA gene [20]. The three polymorphisms (−322G>A, −223G>A, and −43G>T) in 5′ FLR identified in this study were not located in their binding sequences. To evaluate the effect of the combination of the polymorphisms in 5′ FLR on transcriptional activity, the identified haplotype patterns for the three polymorphisms in 5′ FLR were analyzed by the luciferase assay. The haplotype patterns did not show significant differences in transcriptional activity, suggesting that the CatA genetic polymorphism in 5′ FLR does not affect transcriptional activity.

The CatA-stable expressing FLP-in 293 cells revealed that variant 3 showed little expression of mature CatA, whereas variant 2 tended to show higher expression of mature CatA than variant 1. Variant 3 expressed the CatA precursor most strongly among the variants, suggesting that variant 3 did not transform from the precursor to mature CatA protein. The lack of a mature form of variant 3 was consistent with the lack of metabolic activity of TAF in the variant 3-expressing cells. A part of exon 4 of the CatA gene was suggested to be a site that interacts with β-galactosidase in the dimerization of CatA [20]. The lack of exon 4 in variant 3 may be responsible for the lack of a mature form of variant 3. There is a difference in the length of the signal peptide between the variants: the signal peptide of variant 1 is expected to be 46 amino acids and the signal peptide of variant 2 is expected to be 28 amino acids. The signal peptide usually consists of 25–30 amino acids, including the N-terminal region, hydrophobic core region, and C-terminal region [21]. In bacteria, the longer N-terminal sequence of the signal peptide compared with the short one reduced the interaction between the signal peptide and signal recognition particle, resulting in reduced efficiency of transport to the endoplasmic reticulum [22]. In the human Fc receptor like A (FCRLA) gene, isoforms which differ by 6 amino acids in the N-terminal sequence of the signal peptide showed different efficiencies of transport to the endoplasmic reticulum [23]. The difference in expression of mature CatA in variants 1 and 2 may be due to the length of the signal peptide.

The polymorphism in exon 2 (85_87CTG>-) was non-synonymous and showed a high frequency in Japanese. Variant 2 with Leu8 showed significantly increased mature protein expression (Figure 4B) and significantly higher drug metabolic activity (Figure 5) compared with Leu9. Leu repeats in CatA were located in the hydrophobic core of the N-terminal sequence of the signal peptide [10]. The length of the N-terminal sequence of the signal peptide and number of Leu repeats have been reported to affect the efficiency of transport to the endoplasmic reticulum in the human carnosine dipeptidase 1 gene, which encodes a serum carnosinase [24]. Differences in the number of Leu repeats in the CatA gene may have affected CatA trafficking to the endoplasmic reticulum and expression of the mature protein. The genetic polymorphisms in exons 2 and 3 were synonymous, suggesting that there is little effect on the function of CatA. Mutations in introns 9, 10, and 11 identified in this study are unlikely to affect splicing because they are located far from the exon-intron boundary. Genetic polymorphisms in intron 7 have been reported to affect the splicing of CatA in GS patients [16], but genetic polymorphisms in introns, such as those responsible for splicing abnormalities, were not detected in the genome of healthy adults in this study.

TAF is a prodrug metabolized by CatA in leukocytes, the site of its pharmacological action, and had pharmacological effects as TFV diphosphate through three steps of metabolism [25]. Therefore, a decrease in the expression level of CatA may cause a decrease in drug efficacy. The medical package insert of TAF states that the concomitant use of telaprevir, a hepatitis C drug with a strong ability to inhibit CatA, is contraindicated, indicating the importance of CatA metabolic activity. In HIV treatment, it is very important to maintain the drug concentration at the site of drug efficacy in order to prevent the generation of resistant virus. For exon 2 polymorphism (85_87 CTG>-) in healthy Japanese subjects, homozygosity was the most common, involving 29 of 48 subjects, suggesting that there were few Japanese subjects with low metabolic activity. It is considered necessary to confirm the amount of CatA protein in the leukocytes of healthy Japanese subjects with exon 2 polymorphism (85_87 CTG>-) in order to further clarify the influence of CatA polymorphism in vivo.

## 5. Conclusions

The major transcripts of the CatA gene in blood cells were found to be variants 1 and 2. Genetic polymorphism in healthy Japanese subjects was detected in the region related to the regulation of CatA protein expression. This genetic polymorphism may affect the drug-metabolizing activity of CatA in vivo, suggesting that it is important information to promote the appropriate use of drugs.

## Figures and Tables

**Table 1:** Oligonucleotide primers for real-time PCR of Cathepsin A variants and GAPDH.

Location	Primer (5′ to 3′)		Primer Lengh (bp)	Product Length (bp)
Total	Forward	CAGATTGCCGGCTTCGTGA	19	116
	Reverse	AAGCGGGAGAACATGGTGAAG	21	
Variant 1,2	Forward	CCCTGGAGTACAACCCCTAT	20	128
	Reverse	CTGGGCGACCTCAGTGTCAT	20	
Variant 2	Forward	CCCGGGATCGATGATCCGAG	20	164
	Reverse	CGGAGTACTGGCGGAAAGACG	21	
Varinat 3	Forward	AGCATGGCCCCTTCCTGATT	20	111
	Reverse	GGGCGACCTCAGTGTCATTA	20	
GAPDH	Forward	ATCAAGAAGGTGGTGAAGCAG	21	95
	Reverse	TCGCTGTTGAAGTCAGAGGAG	21	

**Table 2:** LC/MS/MS condition for the analysis of TFV-Ala.

LC	LC:		Nexera (Shimadzu)			
	Column:		Mastro C18, 3 μm, 2.1 × 150 mm (Shimadzu GLC)			
	Column temp.:		35 °C			
	Auto sampler temp:		4 °C			
	Mobile phase A:		10 mM Ammonium bicarbonate			
	Mobile phase B:		Acetonitrile (MeCN)			
	Weak wash:		5% MeCN			
	Strong wash:		MeCN/MeOH/IPA/H_2_O = 1/1/1/1 (*v*/*v*), 0.2% formic acid			
	Gradient					
	Time (min)		Flow rate (mL/min)	A (%)		B(%)
	0		0.4	98		2
	1.5		0.4	98		2
	5		0.4	5		95
	6.5		0.4	5		95
	6.6		0.4	98		2
	8		0.4	100		stop
MS	Mass spectrometer:		LCMS8050			
	Scan type:		MRM			
	Ionization:		ESI			
	Polarity:		Positive			
	Interface voltage:		4 kV			
	Nebulizing gas:		3 L/min			
	Heating gas:		10 L/min			
	DL temperature:		250 °C			
	Heat block temperature:		400 °C			
	Drying gas:		10 L/min			
	MRM condition					
	Analyte	Q1 *m*/*z*	Q3 *m*/*z*	Q1 pre bias	CE	Q3 pre bias
	TFV-Ala	359.0	270.0	−26.0	−20.0	−20.0
	Daidzein (IS)	255.1	199.1	−17.0	−25.0	−22.0

MRM: Multiple Reaction Monitoring, CE: Collision Energy.

**Table 3:** Cathepsin A genetic variations in healthy Japanese volunteers.

Location	CDS Position *	Reference Number	V/R Allele	Amino Acid Substitution	*n*	Number of			Frequency of Variant Allele (%)
						R/R	V/R	V/V	
Exon 1	−322	rs2868362	G>A	-	76	25	33	18	42.11
	−223	rs117529875	G>A	-	76	74	2	0	1.32
	−43	rs116893852	G>T	-	76	54	19	3	16.45
Exon 2	85_87	rs72555383	CTG>-	29 Leu >-	48	2	17	29	78.13
	108	rs181943893	G>C	Synonymous	48	39	6	3	12.5
Exon 3	273	rs742035	C>G	Synonymous	48	39	9	0	9.38
Intron 9	924-19	rs3215446	C>-	-	48	14	19	15	51.04
Intron 10	1002 + 7	rs2075961	G>A	-	48	0	13	35	86.46
Intron 11	1142 + 10	rs4608591	C>T	-	48	0	13	35	86.46

*n* = 48 or 76, CDS:coding sequence, R:reference allele, V:variant allele, * With respect to the translation start site of Cathepsin A gene; A in ATG is designated +1, Reference allele: Genbank accession no. NM_000308.4.

**Table 4:** Haplotypes of Cathepsin A 5′-FLR.

Haplotype Pattern	Genetic Variations			Estimated Frequency (%)
	−322G>A	−223G>A	−43G>T	
#1	A	G	G	45.4
#2	G	G	G	37.0
#3	G	G	T	16.3
#4	G	A	G	1.18
#5	A	G	T	NE

Estimated frequency was calculated by Arlequin V3.5. NE: Not estimated.

## Data Availability

Not applicable.
